# Delayed Pneumocephalus-Induced Cranial Neuropathy

**DOI:** 10.1155/2013/105087

**Published:** 2013-09-18

**Authors:** Neena I. Marupudi, Monika Mittal, Sandeep Mittal

**Affiliations:** Department of Neurosurgery, Wayne State University, Detroit Medical Center, and Karmanos Cancer Institute, 4160 John R Street, Suite 930, Detroit, MI 48201, USA

## Abstract

Pneumocephalus is a common occurrence after cranial surgery, with patients typically remaining asymptomatic from a moderate amount of intracranial air. Postsurgical pneumocephalus rarely causes focal neurological deficits; furthermore, cranial neuropathy from postsurgical pneumocephalus is exceedingly uncommon. Only 3 cases have been previously reported that describe direct cranial nerve compression from intracranial air resulting in an isolated single cranial nerve deficit. The authors present a patient who developed dysconjugate eye movements from bilateral oculomotor nerve palsy. Direct cranial nerve compression occurred as a result of postoperative pneumocephalus in the interpeduncular cistern. The isolated cranial neuropathy gradually recovered as the intracranial air was reabsorbed.

## 1. Introduction

Pneumocephalus, defined as air in the cranial vault from pathological communication with extracranial ambient air, is almost always noted following supratentorial craniotomy and is usually asymptomatic [1]. On occasion, however, patients may develop severe headaches with rare concurrent development of neurologic manifestations. Intracranial air along the course of a cranial nerve can result in isolated cranial nerve palsy from direct compression. A review of the literature revealed only three reports of isolated, single cranial nerve deficits due to pneumocephalus [2–4].

We present a patient who developed isolated cranial nerve deficit from direct neural compression by intracranial air in the interpeduncular cistern following occipital craniotomy for transtentorial interhemispheric approach for resection of a pineal region mass.

## 2. Case Report

**Figure 1:**
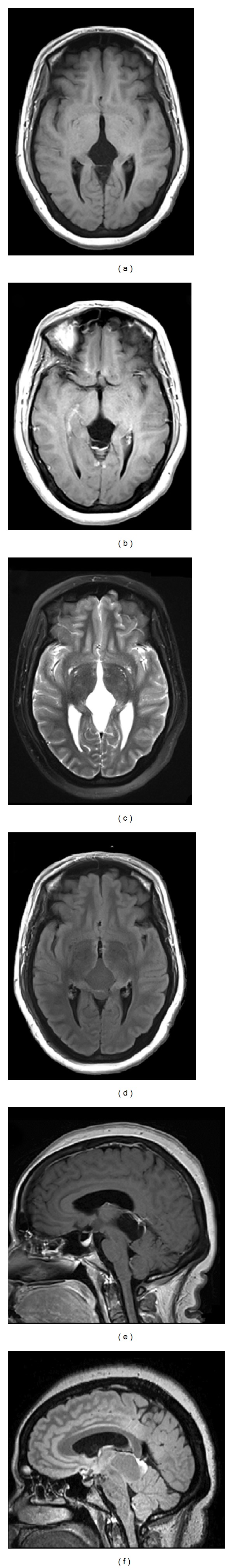
Preoperative MRI findings. Axial T1-weighted image (a) shows a 3.7 × 2.2 × 2.7 cm well-circumscribed complex cystic lesion in the region of the pineal gland, causing significant mass effect on the surrounding structures. There is inferior compression and thinning of the tectal plate, posterior displacement and deformity of the superior cerebellar vermis. T1 postcontrast images ((b) and (e)) show the thin septation posteriorly. There is no enhancement of the cyst wall. On T2-weighted images (c), the signal characteristics are similar to CSF, but the cyst fluid is isointense to slightly hyperintense on FLAIR images ((d) and (f)).

An otherwise healthy 30-year-old woman presented with a five-year history of syncopal episodes, moderate to severe headaches, intermittent diplopia, vertigo, and tinnitus. No focal neurological deficits were noted on examination. CT scan of the head showed a cystic lesion in the pineal region extending into the posterior third ventricle and causing significant mass effect on the midbrain, superior cerebellar vermis, and thalamus. MRI of the brain (Figure 1) demonstrated a 3.7 × 2.2 × 2.7 cm cystic pineal lesion with no enhancement of the cyst wall except for a thin septation seen posteriorly and mild hydrocephalus.

The patient underwent a stereotactic right occipital craniotomy with complete resection of the cystic pineal mass via a transtentorial interhemispheric approach. The procedure was performed under general anesthesia with the patient positioned in the prone position. The surrounding neurovascular structures were identified and carefully preserved including the right basal vein of Rosenthal, right and left internal cerebral veins, vein of Galen, and precentral cerebellar vein. No brain retractors were used during the procedure, and no notable complications were encountered during surgery. Histopathological analysis was consistent with a benign glial cyst of the pineal gland.

**Figure 2:**
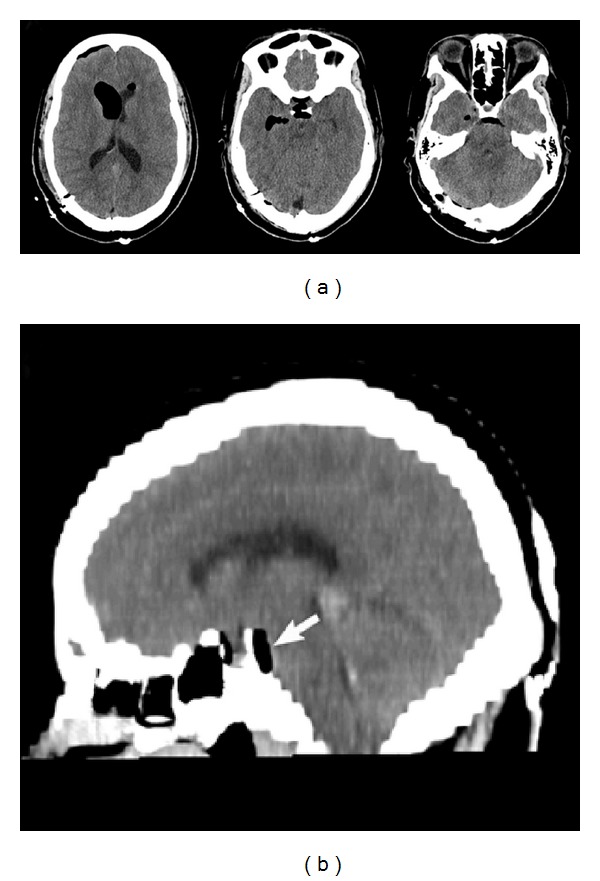
Immediate postoperative CT scan. Postoperative changes following resection of pineal region cystic lesion are evident in conjunction with significant pneumocephalus. Intracranial air is seen diffusely in the subarachnoid and ventricular spaces (a). A significant amount of air is also noted in the interpeduncular and prepontine cisterns. The midsagittal reconstructed image highlights the interpeduncular air (arrow; (b)).

**Figure 3:**
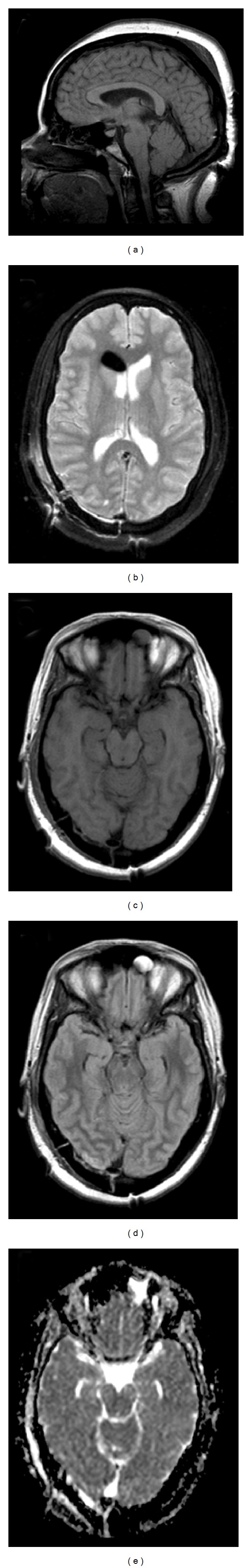
Postoperative MRI obtained within 24 hours after surgery. The midsagittal T1-weighted image shows complete resection of pineal cyst with reexpansion of the tectum (a). There is significantly reduced pneumocephalus in the subarachnoid space, but ventricular air is still present (b). Of note, there is no hemorrhage on T1 (c), edema on FLAIR (d), or ischemic changes on diffusion restriction images (e) in the vicinity of the oculomotor nuclei in the dorsal midbrain or oculomotor nerves in the ventral midbrain.

Postoperatively, the patient awoke from the surgery, was alert and appropriately oriented, and followed commands briskly. In the postanesthesia care unit, she complained of a mild headache but otherwise had a normal neurological examination. However, six hours later, she developed severe dysconjugate gaze resulting in diplopia. She had slightly outward and downward deviation of both eyes, with the left side worse than the right, concerning for bilateral pupil-sparing third nerve deficits. Her remaining neurological examination was otherwise normal. An urgent CT scan was obtained; no intracranial hemorrhage was noted, but there was considerable pneumocephalus in the right lateral ventricle. In addition, the interpeduncular and prepontine cisterns were entirely filled with air (Figure 2). Routine postoperative MRI, done the next morning, confirmed complete excision of the complex pineal cyst with decompression of the third ventricle without evidence of hemorrhage, FLAIR hyperintensity, or diffusion restriction in the tectal plate (Figure 3).

**Figure 4:**
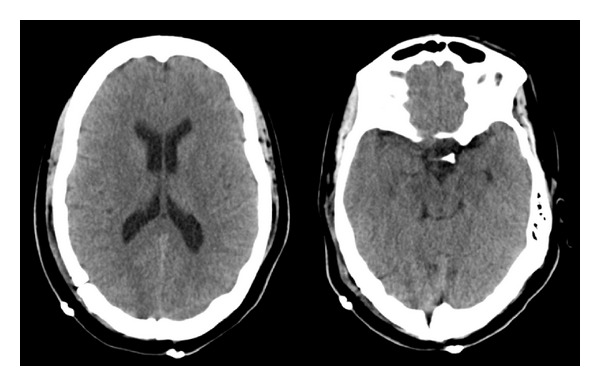
Postoperative CT scan done 2 weeks after surgery showing complete resolution of pneumocephalus.

Although tectal tumors are often associated with postoperative Parinaud's syndrome, given the patient's otherwise normal light perception, accommodation on convergence, and overall lack of pupillary dysfunction, Parinaud's syndrome was excluded. One week later, a CT scan showed resolving pneumocephalus. Likewise, the patient also had improved ophthalmoplegia with near complete normalization of her left eye movements, although partial oculomotor nerve palsy was still noted on the right side. Follow-up CT scan 2 weeks later demonstrated complete resolution of the pneumocephalus (Figure 4). The patient's oculomotor nerve palsy also improved considerably with complete resolution in both eyes noted at the 3-month postoperative follow-up visit. She remained neurologically intact without evidence of recurrence of the cystic mass on MRI 3 years after surgery.

## 3. Discussion

Although pneumocephalus is almost invariably seen following cranial surgery, the development of focal neurological impairment from the presence of intracranial air is rare. Isolated cranial nerve deficits resulting from direct compression by intracranial air are even more uncommon. In fact, there are only three prior reports of patients who developed cranial neuropathy due to pneumocephalus. We highlight a case of bilateral pupil-sparing oculomotor nerve palsy as a result of direct neural compression from postsurgical development of pneumocephalus in the interpeduncular cistern. This is further supported by the fact that the clinical symptoms were delayed and resolved in conjunction with resolution of the pneumocephalus on serial neuroimaging. No brain retractors were used during the dissection, and there was no direct or indirect traction placed on the involved cranial nerves or nuclei. Postoperative Parinaud's syndrome was excluded. Furthermore, on postsurgical MRI, there were no signs of edema or ischemia in the vicinity of the oculomotor nuclei on T2, FLAIR, or diffusion-weighted images.

Similar to our patient, two other cases of oculomotor nerve palsy have been reported from direct compression from pneumocephalus. Laviola et al. described a 27-year-old woman who developed severe bifrontal headache and a left-sided unilateral pupillary dilatation consistent with third nerve involvement after an incidental dural puncture during epidural anesthesia for cesarean section [2]. CT scan of the head demonstrated subarachnoid air in the anterior portion of the interpeduncular cistern. The authors suggested that the left pupillary dilatation was the result of direct pressure on the ipsilateral oculomotor nerve from the intracranial air [2]. The patient's symptoms resolved after 6 days. Aygun et al. reported a 23-year-old man who developed a bilateral third nerve palsy after acute head trauma resulting in a depressed skull fracture [3]. In this case, the isolated oculomotor nerve deficits were attributed to perimesencephalic pneumocephalus resulting in involvement of the third nerve nucleus. This patient also was noted to have resolving symptoms associated with resolution of the intracranial air [3]. 

Stevens et al. reported a 13-year-old girl who developed unilateral lateral rectus palsy postoperatively following resection of a pineal cyst in the sitting position [4]. Interestingly, her symptoms of an isolated sixth nerve palsy developed four days after surgery. The authors hypothesized that the delayed unilateral abducens palsy was a result of caudal displacement of supratentorial structures by the postoperative pneumocephalus resulting in indirect traction or compression on the sixth cranial nerve [4].

The oculomotor and abducens nerves are amongst the most resilient cranial nerves, requiring considerably higher forces to result in functional impairment [5]. Several mechanisms exist by which intracranial air can develop enough disruptive strength to cause direct cranial nerve compression. One mechanism that can lead to intracranial air is the ball-valve system whereby ambient air can enter through a cranial defect following, for example, a Valsalva maneuver. Subsequent shifts of the brain tissue and/or dura secondary to raised intracranial pressure (ICP) from the forced air can ultimately result in the air being trapped in the cranium [3, 6]. Another manner by which postoperative pneumocephalus can develop is the low (or sometimes negative) ICP that occurs following intraoperative loss of CSF and diuresis. This creates a pressure gradient leaving potential spaces and negative pressure, thereby allowing air to occupy the space once the brain reexpands [7, 8]. This mechanistic phenomenon can be demonstrated following clamping a CSF drain that allows for the buildup of fluid and resolution of pneumocephalus. Conversely, restarting the flow or external drainage of CSF again results in recurrence of pneumocephalus [6]. One factor that can exacerbate the development of pneumocephalus is increasing body temperature, which causes expansion of air and may result in symptoms of acute compression of surrounding neurovascular structures [8]. Based on these mechanisms, air trapped in the cranium, especially when it enters and becomes trapped within a cisternal subarachnoid space, can expand the cistern and consequently develop enough force to compress a cranial nerve along its trajectory within that space. Considering the mechanism of pneumocephalus development and the small area of the potential spaces that the cranial nerves traverse through, even a small amount of trapped air would result in enough compressive force to affect the respective cranial nerves.

Certainly, pneumocephalus is quite common following craniotomy, and this case on initial inspection demonstrates the normal amount of pneumocephalus that would be expected from surgery. It is also true that excessive CSF drainage and the resulting traction on the brain could result in cranial neuropathies as well; however, the surgical procedure did not result in loss of any unusual amounts of CSF that would have resulted in traction on the brain or associated cranial nerves. Furthermore, our patient demonstrated a delayed presentation of her neuropathy excluding surgical sequelae and supporting a mechanism of pneumocephalus development where air entered the cisternal spaces over several hours postoperatively, resulting in a compressive force on the third cranial nerves.

The clinical course and neuroimaging findings associated with pneumocephalus-induced isolated cranial neuropathy are self-limiting, and full recovery is expected.
